# Investigating the Time Course of Part-Based and Holistic Processing in Face Perception

**DOI:** 10.3389/fpsyg.2018.02630

**Published:** 2019-01-17

**Authors:** Chao-Chih Wang

**Affiliations:** Research Center for Education and Mind Sciences, Hsinchu Teachers College, National Tsing Hua University, Hsinchu, Taiwan

**Keywords:** face perception, part-based processing, holistic processing, time course, composite face illusion, dual-route model

## Abstract

Human has an exceptional ability for face recognition to keep up social network. However, it is unclear to understand the mechanisms of face recognition until now. Specifically, there is less research to examine the time course of part-based and holistic processing when these two routes trigger and finish. In the present experiments, the exposure time was manipulated to examine the time course of face processing and found evidence suggesting that holistic processing occurs shortly after part-based processing at about 200 ms, and can last for a relatively long duration up to 2,000 ms. These results may support to a dual-route model comprising holistic processing and part-based processing in face perception. Moreover, our findings were inconsistent with the previous study which suggests that no holistic processing was observed at the relatively long duration, and suspected that perceptual discriminability may have been responsible for the discrepancy.

## Introduction

**FIGURE 1:**
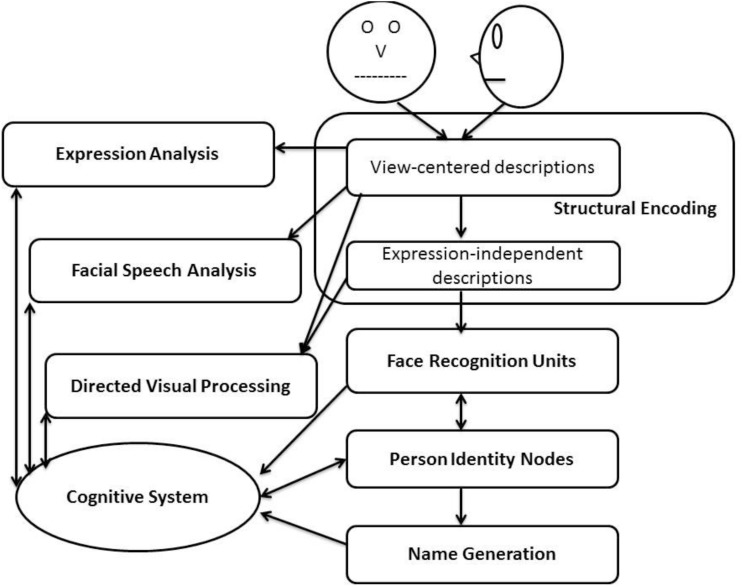
The functional model of familiar face recognition (re-drawn from Bruce and Young, 1986).

Ever since Galton’s (1879) insight that face recognition is most likely based on integration of facial features, holistic processing has been construed as a very important component to understand how humans perceive faces (Young et al., 1987; Tanaka and Gordon, 2011). Face perception is a distinct human ability for recognizing members of your family, friends and foes, celebrities and enemies, even caricatures and cartoons. The same level of ease and efficiency of face recognition performance is difficult to meet even with the enormous information-processing and computational power of advanced computers today, largely because the underlying processing seems so complicated that the proper level of algorithms for achieving the task is not clear for engineers (Richler et al., 2012). Although humans could recognize thousands of people, oftentimes flawlessly, many questions remain as to how this unusual ability of face recognition is achieved. In their seminal and influential article, Bruce and Young (1986) proposed the first *functional* model to explain how human recognizes familiar faces (see Figure 1). According to Bruce and Young, recognizing familiar faces entails a match between information of structural encoding of the face that is present and stored codes of face recognition units (FRUs). Subsequently, identity-specific semantic codes are processed and created from person identity nodes and name codes are retrieved. Cognitive system plays the role of deciding whether or not the initial match is adequately close for veridical recognition or only a degree of likeness, and takes into account a number of factors before reaching the final decision.

Bruce and Young’s (1986) functional model has been credited for accounting for many aspects of (familiar) face processing in terms of the relevant codes. Although structural encoding may well comprise both part-based processing and configural processing, Bruce and Young did not explicitly make that distinction. On the other hand, Maurer et al. (2002) had proposed that there are three types of configural processing: detecting the presence of first-order spatial relations that define a face, holistic processing that integrates facial features into a gestalt whole, and processing of second-order spatial relations that is sensitive to spacing and metric information among facial features. McKone (2010), however, contended that there is no convincing evidence for dissociation or separation between holistic processing and processing of second-order relations. Instead, she argued that there are many theoretical reasons to consider holistic processing to be conducted on a single integrated representation, which she would dub as holistic/configural processing, rather than comprising a number of separate subcomponents (McKone, 2010). As yet another alternative, there could be a compromise between the view espoused by Maurer and her colleagues on one hand and that by McKone on the other. That is, the processing of face recognition system may be divided into two subcomponents, comprising face detection and holistic face processing, because there is convincing evidence to support the separation between these two parts (Maurer et al., 2002; McKone, 2010).

**FIGURE 2:**
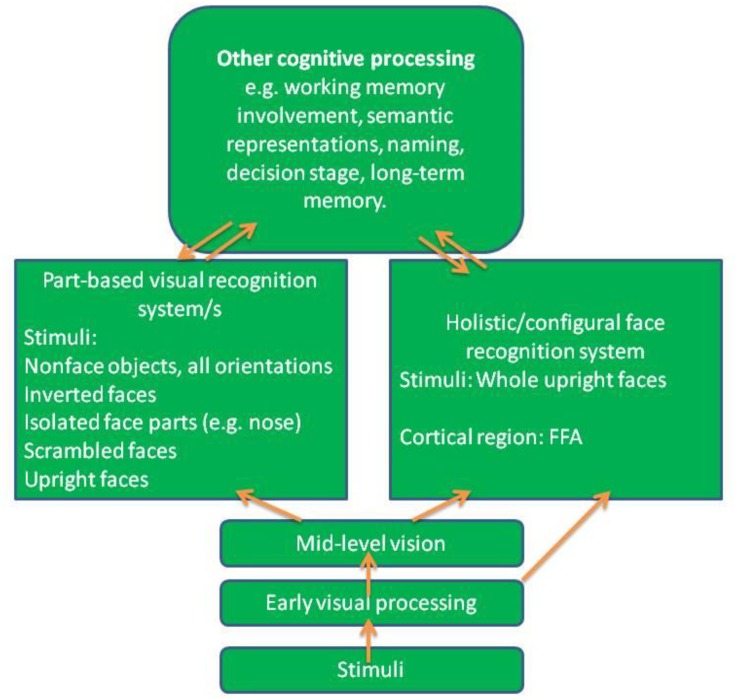
Early visual processing, mid-level vision and long-term memory all would affect holistic processing with the exception of part-based visual recognition system (re-drawn from McKone, 2010).

Moreover, McKone had proposed a dual-route model for face processing where holistic/configural face recognition system and part-based visual recognition system operate independent of each other concretely. According to McKone (2010, see also McKone and Yovel, 2009), holistic processing comprises extraction of a variety of information from a face, including spacing between centers of major nameable facial features such as interocular distance, nose-mouth distance, as well as overall position of features in a face, while excluding shape details of local features. As illustrated in Figure 2, McKone (2010) suggested that holistic/configural face recognition system diverges from part-based visual recognition system after the point where face processing has gone through mid-level visual processing. After that, the products of mid-level processing are fed into two parallel routes for further processing, representing, respectively, the part-based visual cognition system and the holistic/configural recognition system. Neuropsychological evidence from patient studies seems to support that there are dissociations between part-based visual recognition system and holistic/configural face recognition system. For example, patient CK suffered from object agnosia and dyslexia as a consequence of a closed-head injury, but was intact for face recognition (Moscovitch et al., 1997). In contrast, McNeil and Warrington (1993) reported that the patient WJ exhibited severe prosopagnosia (impaired ability in face recognition) but had intact ability in object recognition. Moreover, some studies indicated that right occipital face area and fusiform face area play an important role for holistic processing (Kanwisher et al., 1997; McKone, 2010; Jonas et al., 2012, 2014; Bona et al., 2016).

What exactly are the differences between part-based visual recognition system and holistic/configural face recognition system? There is a main issue needs to be addressed concerns whether or not the part-based processing can trigger holistic processing such that albeit structurally separated their functional operations are related or whether they are strictly independent of each other with no possibility of functional links as McKone had proposed (see Figure 2). Thus far, the evidence has been inconsistent: For example, Amishav and Kimchi (2010) suggested that both componential and configural processing are integral for upright faces because participants could not selectively perceive features without simultaneously being influenced by configurational variation and vice versa. Their findings appear to implicate that holistic processing and part-based processing did not operate independently. On the other hand, Fitousi (2015, 2016) employed the system factorial technology (SFT) as a means for mathematical modeling of the nature of face processing, and argued that face recognition is achieved analytically rather than holistically. Specifically, he was able to demonstrate the existence of composite face effect, but failed to obtain Garner interference and super-capacity with the same composite faces. These results are consistent with analytic processing (Fitousi, 2015, 2016). Therefore, further studies are required to help the controversial issue.

There are at least three important questions need to be answered with regard to McKone’s (2010) dual-route model: first, as discussed earlier, if holistic/configural face recognition system is unitary rather than comprising subcomponents, how would the dual-route model explain the fact that people who suffer from prosopagnosia still can detect the presence of a face, while fail to recognize familiar faces? If people afflicted with prosopagnosia can nevertheless detect the presence of a face, it would imply that there is dissociation between face detection and holistic processing. Second, is there evidence to support the hypothesis regarding the time course to demonstrate separation between part-based processing and holistic/configural processing? If the results suggest temporal differences in the operation of these two modes of processing, that would provide credence to support the separation between part-based visual recognition and holistic/configural face recognition systems. On the other hand, if the results do not suggest temporal differences in the operation of these two modes of processing, it does not necessarily implicate whether these two modes of processing are interactive or whether they can operate independently. In other words, logically speaking, the difference in time course is a sufficient condition but not necessary condition for arguing the separation between part-based and holistic/configural holistic processing. Moreover, the separation between two modes of processing does not necessarily predict differences in time course. For example, the part-based processing and holistic processing can operate in parallel and simultaneously with no temporal difference, as seems to be implied by McKone’s model depicted in Figure 2. Finally, both the part-based visual recognition and holistic/configural face recognition systems are temporally subsequent to the mid-level vision. However, it is not clear how and what kind of information from the mid-level visual processing would be funneled into the separate routes of the part-based processing and holistic/configural processing.

Regarding the first question, McKone had suggested prosopagnosic patients could rely on part-based recognition system to detect presence of faces, whereas damage to the holistic/configural face recognition systems would prevent them from recognizing familiar faces. Consistent with this interpretation, a number of studies had demonstrated that indeed prosopagnosia patients can detect faces, and at the same time showed impairments in holistic processing (e.g., Avidan et al., 2011). Regarding the second question, it seems that there can be no clear-cut temporal separation between part-based processing and holistic processing because holistic processing occurs rapidly within 50 ms after the onset of face stimuli (Richler et al., 2009). In other words, findings from previous behavioral studies have offered little evidence for the temporal separation between holistic processing and part-based processing. Finally, regarding the third question, while making assumptions about the separate, parallel routes of processing for her dual-route model, McKone failed to give sufficient details to subject the proposed model to empirical enquiries, and factors that putatively would give rise to holistic processing were also left unspecified. Regardless whether holistic/configural face recognition system is unitary or comprises separate subcomponents, it is important to account for how the product of mid-level vision would channel face processing into part-based recognition systems and holistic/configural face recognition systems in general, and how exactly holistic/configural processing can be derived from the mid-level vision in particular. One likely candidate for the product of mid-level vision would be a representation akin to the face template, and many studies have suggested that holistic processing arises from a face template (Gauthier et al., 2010; Richler et al., 2012; Richler and Gauthier, 2014). However, there has been very little research to examine directly the relationship between a face template and holistic processing.

According to Richler et al. (2012), many putative mechanisms can be hypothesized to be potential instigator for holistic processing, including global face templates, representation of spatial relations between parts, inflexible attentional weightings on parts and parallel coactive processing, etc. It would require a formidable amount of studies to test the validity of each of these hypothesized mechanisms. Even though many researchers have indicated that face parts are recognized holistically as a gestalt based on a face template (e.g., Young et al., 1987; Tanaka and Farah, 1993; Farah et al., 1998), the idea of face template remains a relatively vague concept for understanding face processing. The following section discuss the relationship between face template and holistic processing.

### Face Template and Holistic Processing

As mentioned above, many researchers seem to have adopted the view that holistic processing arises as a consequence of extensive experiences with a face template (Tanaka and Farah, 1993; Rossion and Boremanse, 2008; Chua et al., 2014). Rossion and Boremanse (2008), for example, found that the composite face illusion, where holistic processing is indexed by the composite face effect (CFE), was equally strong when faces were presented at 0°–60° on a fronto-parallel picture plane, then dropped off at 90° and maintained up to 180°. They contended that faces have been processed holistically at angular rotation less than 90°, and the dramatic drop of composite effect when faces were rotated beyond 90° suggests that holistic face perception may have resulted from experience-driven template of upright faces (Rossion, 2013).

However, other researchers have suggested that face parts are “glued” together to form a unitary perceptual representation or “face template” (e.g., Young et al., 1987; Tanaka and Farah, 1993; Farah et al., 1998). Such usage of terminology and descriptions can sometimes be confusing and misleading because it leaves very little room for distinction between face template and holistic processing. As a more useful alternative, consider Tsao and Livingston’s (2008) proposal that face detection is the first step in face processing, especially when the face is embedded in a complex visual scene. Importantly, their proposal implicates that a face template can be based on the simple T-shape configuration comprising a pair of eyes above a nose and the nose above a mouth. Neurophysiological evidence has led credence to such a notion of face template, where many studies of single-cell recording have found that face cells require an intact face configuration as effective eliciting stimulus, and are not selectively responsive to facial features (Bruce et al., 1981; Desimone et al., 1984; Kobatake and Tanaka, 1994; Tsao et al., 2006). These studies demonstrated the dissociation between an intact face configuration (i.e., face template) and facial features comprising the configuration.

In addition, activation in inferior temporal cortex for face-selective neurons begins firing 80–130 ms after stimulus onset (Bullier, 2001). An ERP study also reported that the time window for face detection was about 130 ms (Jacques and Rossion, 2006). Furthermore, according to Bentin et al. (1996), an inverted face and partial facial features (e.g., nose and lips) had a delayed N170 component in comparison to an upright face, which implies different time courses for holistic processing and part-based processing. However, there have been relatively few behavioral studies that investigate the time course difference between holistic processing and part-based processing, and in particular it is unclear whether holistic processing arises from a face template directly (Chua et al., 2014).

It should be noted that there are at least two different notions of face template being advocated in the existing literature. On one hand, face template and holistic processing are treated as synonymous and have been used interchangeably; on the other hand, face templates strictly entail the T-shape configuration and first-order relational properties within a face.

### Holistic Processing and Sensitivity to Configuration

**FIGURE 3:**
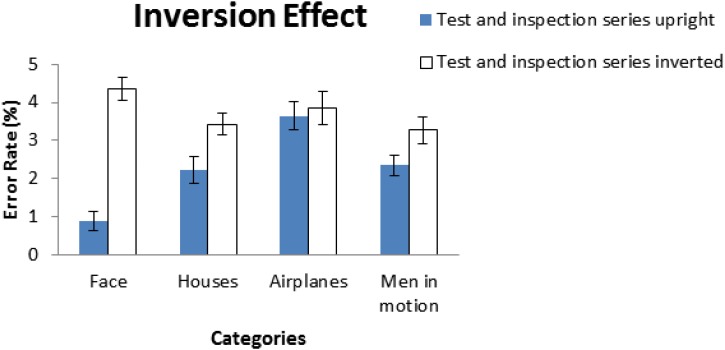
In Yin (1969) study, there was a significant difference in performance between upright and inverted faces; in contrast, no significant differences were found for objects of non-face categories. The error bar indicates the mean of ±1 standard error. The figure was adapted and modified from Yin (1969).

Yin’s (1969) study was the first that demonstrates the disproportionate inversion effect with faces. During both the perception and memory phase of his study, Yin’s participants showed better performance with upright faces than with inverted faces (Figure 3). In a sharp contrast, inversion had little effect on non-face objects such as houses and airplanes. Based on these findings, Yin suggested that perception of human face is strongly influenced by orientation, due to the fact that faces were processed holistically and inversion disrupts holistic processing and results in poorer performance on inverted faces.

Subsequently, Diamond and Carey (1986) proposed that three conditions need to be met to lead to performance decrement for the inversion effect: first, members of the group of stimuli must share the same first-order configuration. Second, differences between individual members are on the basis of second-order relational features. Finally, participants who are experts can distinguish these members of the group relying upon such second-order relational information. As a case to support their conjecture, Diamond and Carey showed in their study that dog experts exhibited inversion effect in recognition of breeds they were most familiar with (and hence showing expertise).

To further examine holistic processing in faces and objects, Tanaka and Farah (1993) used a part-whole task and showed that people can better recognize a face part (e.g., nose) in the context of a whole face than in isolation. In contrast, they found no part-whole differences when participants were asked to recognize other kinds of object such as scrambled parts, inverted faces, and houses.

Tanaka and Farah argued that their findings suggest people recognize faces by engaging in holistic processing. Moreover, in a subsequent study, Tanaka et al. (1998) argued that children develop holistic processing for faces by the age of 6 years old, although more generally, face recognition seems to mature at around 12 years of age (Johnson and Morton, 1991; Mondloch et al., 2002). Studies in perceptual grouping also suggest that around 5–6 years old, children have developed perceptual organization capability comparable to that for adults (Hadad and Kimchi, 2006). It should be noted, however, it is unclear whether holistic/configural face recognition system is different from part-based recognition systems because there is a lack of relevant evidence to support the dual-route model based on developmental studies.

**FIGURE 4:**
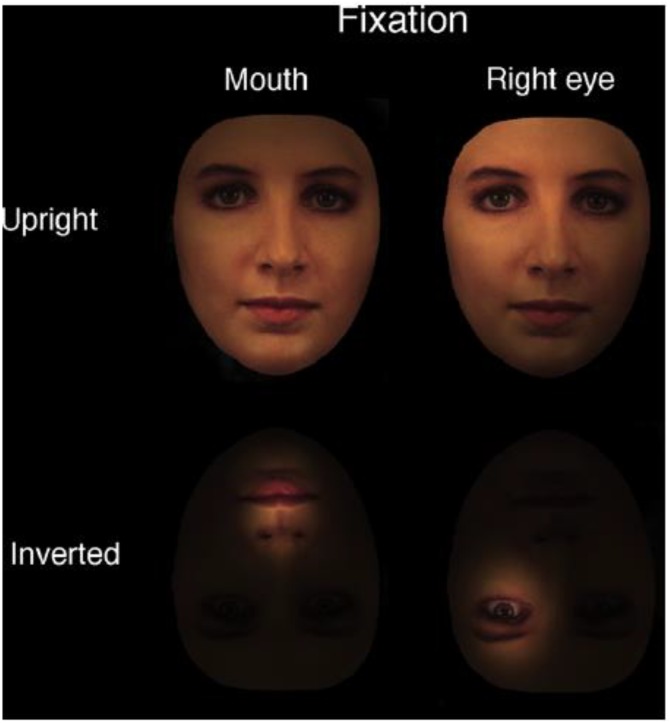
According to the perceptual field hypothesis, participants perceive an upright face as a whole face, rather a collection of local features. In contrast, participants perceive inverted faces as local features, rather than a whole face. The figure was adapted from Rossion (2009) with permission.

Although disruption of holistic processing has been hypothesized to account for inversion effect, it is less clear what exactly may have taken place during inversion that would lead to such disruption. One possible candidate for the underlying mechanism is the perceptual field hypothesis Rossion (2009) proposed, which would explain how inversion impairs holistic processing in the following manner: upon seeing an upright face, observers presumably would expand their perceptual field to the fullest extent in order to perceive the whole face (Tanaka and Gordon, 2011; Rossion, 2013). In contrast, when seeing an inverted face, observers would perceive something less than a whole face, which likely is composed of a constellation of local facial features due to the contraction of perceptual field (Figure 4). It is interesting to note that McKone (2010, p. 284) also mentioned in the passing a similar account for holistic processing arisen from “a big receptive field” being applied to the entire face.

### Composite Effect and Composite Face Illusion

**FIGURE 5:**
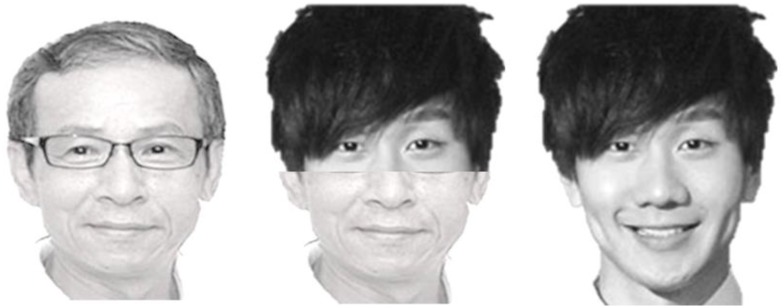
The face in the middle, which is a composite made of top half of the face on the right (Jun Jie Lin) and bottom half of the face on the left (Wen-Ching Wu, though professionally he goes with name Nian-Zhen Wu), was similar to those used by Young et al. (1987), where the top and bottom halves of faces were drawn from two different celebrities to form a novel composite face. The right face is a famous singer in Taiwan (Jun Jie Lin) and the left face is a writer-turned-celebrity in Taiwan and China. When naming the composite faces based on part of the faces (e.g., top halves), participants’ performance was easily interfered by the irrelevant bottom part when the two halves were aligned, but not as much when they were misaligned.

Young et al. (1987) developed another paradigm—the composite faces—to demonstrate holistic processing of faces (Figure 5). Although they used the term “configural” rather than “holistic,” to avoid conceptual confusion, it would consider their effect as demonstrating holistic, rather than configural, processing because composite faces involve altering both featural and configural information of a face simultaneously. In their experiments, Young et al. created composite faces of celebrities in that the top and bottom half parts of different celebrities were joined to form novel faces. Participants’ performances in naming the celebrity based on top half of the face were severely compromised by the creation of composite faces. Young et al. concluded that participants recognized famous faces by means of holistic processing. Following Young et al’s. (1987) study, composite effect is not obtained with objects or non-face stimuli (Robbins and McKone, 2007), in addition to the fact that they were little affected by inversion.

Following Young et al.’s study, Hole (1994) further demonstrated that unfamiliar faces also were affected by the irrelevant parts. In each trial of their experiment, a pair of faces was simultaneously presented, which was different from the naming task used by Young et al. (1987). Despite the difference in task requirement, Hole’s findings were consistent with those reported by Young et al. (1987). Taken together, these studies suggested that both familiar and unfamiliar faces are processed holistically rather than in a piecemeal manner (Young et al., 1987; Tanaka and Farah, 1993; Hole, 1994).

Ever since the pioneering work by Young et al. (1987) and Hole (1994), many subsequent studies have adopted the composite face task to assess the failure of selective attention to the target part, which was interfered by the irrelevant parts as a result of combining halves from different faces. Participants cannot focus selectively on the specific part of a face (e.g., the top half) while at the same time ignore the irrelevant part (e.g., the bottom half), implicating that face recognition is achieved via integrated holistic processing, rather than via a piecemeal process where each facial feature was processed and then combined. However, as Rossion (2013) pointed out, there is a difference between composite effect and composite face illusion. Specifically, he argued that participants actually perceived the composite face as a new face, in the sense that facial features had been modified perceptually rather than being perceived veridically (*called* composite face illusion). As a result, holistic processing for upright faces not only affects sensitivity of configuration among features but also on the features themselves. Maybe, it is better to the term *composite face illusion* rather than composite face effect.

### A Note on Conceptual Clarification and the General Method

Before describing the experiments proper, I would like to clarify the conceptual definitions of a number of popular notions to avoid the possible confusions (cf. Richler et al., 2012). In so doing, it is necessary to give a description of the General Method used here.

Generally speaking, *holistic* processing of a face refers to integral processing of all parts of a face as a whole in that processing one part of the face would inevitably entail processing another part of the face. More specifically, there are two important and yet distinct aspects regarding the notion of holistic processing. The first aspect concerns the perceptual integrality just mentioned. The second aspect concerns the strong and inherent dependency of orientation in face processing where holistic processing is more evident and robust for an *upright* than for an inverted face. *Part-based, featural* or *component* processing of a face, on the other hand, typically involves manipulation or alteration of face components while keeping intact the configuration and the metrical distances between components (Mondloch et al., 2002). Finally, *configural* processing of a face entails manipulation of inter-distance between face components while keeping intact the components (Gauthier and Tarr, 2002; Mondloch et al., 2002).

As Rossion (2008) pointed out, although configural processing and holistic processing can be dissociated conceptually, they are confused inherently in the practice of empirical enquiry. While most researchers would agree that *holistic* processing for face recognition entails simultaneously integration of multiple features of a face into a single representation (Rossion, 2008), there remains the question whether configural processing should be treated as part of holistic processing. For some researchers, it has been common to associate changes in spacing between facial features with holistic processing (Gauthier and Tarr, 2002; McKone, 2010). For instance, Gauthier and Tarr (2002) treated the term “holistic” as a superordinate concept and encompassed a variety of effects, such as holistic-configural, holistic-inclusive, and holistic-contextual effects. In the view, I would like to define *holistic* processing as the integration of component and configural processing, which is similar to the “holistic-contextual effect” proposed by Gauthier and Tarr (2002).

Previous studies have used definitions that in the view were not completely compatible with one another (Richler et al., 2012). Robbins and McKone (2007), for example, used the term “configural/holistic processing” to suggest that configural processing is synonymous to holistic processing, and it was not necessary to make fine distinction between them given the current state of research findings and evidence (see also McKone, 2010). However, Mondloch et al. (2002) study had argued earlier for the distinction between configural and holistic processing, where configural processing focuses on spatial (metric) relations between and among face parts, and hence is different from holistic processing, which focuses on the integration of face as a perceptual whole or gestalt. Moreover, Hole (1994) used the term, “configurational factor,” to describe the composite-face effect, whereas Gauthier and her colleagues have over the years used the term of “holistic processing” in an all-embracing manner to refer to the processing underlies the composite-face effect. Together these examples highlight the fact that the term “holistic processing” has not been used in a completely coherent and consistent manner in the literature, where not only it has different meanings for different researchers but also may refer to different tasks and different associated effects (Richler et al., 2011).

*Partial* versus *complete* design of composite face task. Gauthier and Bukach (2007) had proposed to use what they called *complete* design to replace the traditional composite task, also called the *partial* design, used in many previous studies based on two reasons. First, some researchers had indicated that only the results from the same trials in the partial design were computed (Robbins and McKone, 2007; Rossion, 2013) while ignoring completely the results from different trials. Gauthier and colleagues argued that the results should include both the same trials and different trials because results from both kinds of trials are meaningful for explaining the composite illusion (Gauthier and Bukach, 2007; Richler and Gauthier, 2014). When different trials are ignored in the partial design, it is impossible to predict whether perceiving information from irrelevant parts facilitate or interfere performance when relevant parts are different. In addition, when the same trials in the congruent condition and different trials in the incongruent condition were missing, there was not enough information to predict how irrelevant part affects holistic processing in those conditions. When the responses for relevant and irrelevant part are the same (i.e., both “same” or both “different”), these trials are in the congruent condition. In contrast, when the responses are different (i.e., one “same” and the other “different” or vice versa), these trials are in the incongruent condition.

The second and perhaps more critical reason is that the partial design is subject to response biases from individual participants (Richler and Gauthier, 2014) because they tend to respond “same” in the upright face condition than in the inverted face condition (Wenger and Ingvalson, 2003; Richler and Gauthier, 2014) and in the aligned condition than the misaligned condition (Gauthier and Bukach, 2007). To rule out the potential problems, Gauthier and Bukach (2007) had proposed that holistic processing should be assessed and measured in terms of a *congruency* effect (i.e., the difference in performance between the congruent and incongruent conditions) and the dependent variable is sensitivity (*d*′), which is a difference score (Z_n_ - Z_sn_) based on signal-detection theory (SDT) (where *n* denotes “noise” condition, and *sn* denotes “signal + noise” condition) (Green and Swets, 1966). The performance in sensitivity (*d*′) is expected to be better on the congruent trials than on the incongruent trials in the aligned (or upright) condition and the magnitude of congruency effect is reduced in the misaligned (or inverted) condition. Moreover, the magnitude of congruency effect with aligned (or upright) faces is expected to be greater than that with misaligned (or inverted) faces. Theoretically speaking, it is appropriate to use the complete design in the composite task.

**FIGURE 6:**
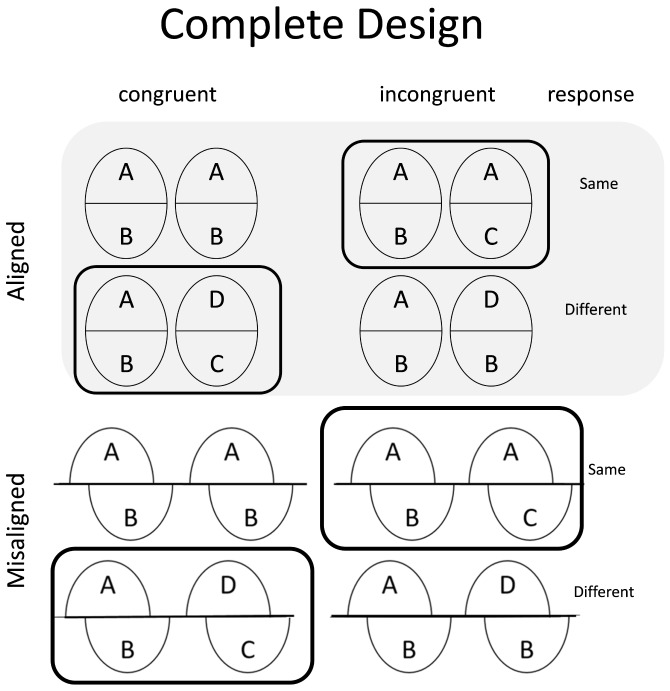
Many prior experiments had used the partial design (black frame) to demonstrate holistic processing, but the irrelevant part was always different. Although it showed slower performances for same trials, it was possible that the poorer performance had resulted from congruency effect, rather than holistic processing. In the complete design, showed at the figure could control the confounding variable and avoid an erroneous conclusion. The figure was redrawn from Gauthier and Bukach (2007).

How can congruency effect be measured? Referring to Figure 6, the complete design involves congruent and incongruent conditions. The congruent condition means that the target and the irrelevant part are both same or both different. In contrast, the incongruent condition means that those are inconsistent. If the present study would calculate congruency effect in aligned condition, the result was that *d*′ of congruent trials minus *d*′ of incongruent trials (Gauthier and Bukach, 2007). Some researchers suggested inverted faces are the same as misaligned face because of impairment for configural processing. On the other hand, the irrelevant parts are always different in the *partial* design (see black framed panels in Figure 6). Moreover, the partial design only calculates accuracy or reaction time between same trials of aligned and misaligned condition and neglect altogether data from different trials.

Moreover, Zhao et al. (2015) recently suggested alignment effect (or interaction between alignment and congruency effect) can even occur for no-face stimulus (e.g., line pattern) as long as it possesses salient Gestalt properties such as connectedness, closure, and continuity between parts. Bases on these observations, Zhao et al. (2015) argued that alignment effect could be achieved not only via a top-down route from becoming experts, but also via a bottom-up route of relying on object-based information.

In addition, McKone et al. (2013) proposed that inverted faces always should be included such that a pure measure of holistic effect can be achieved to the extent that there is an absence of holistic processing with inverted faces. Based on these suggestions, inverted condition could be a better measure to detect holistic processing than the manipulation of alignment. Therefore, in the experiments reported here, the adopted dependent variable was the difference in congruency effect between upright and inverted conditions using the *complete* design.

Besides the definition of holistic processing for the dependent variable, the present study also measured the performances of the part-based processing as the dependent variable in each experiment. According to McKone (2010), the part-based processing includes upright and inverted faces. Therefore, the definition of part-based processing included the performances of the upright and inverted conditions. In other words, the performances of part-based processing were the sensitivity for the upright and inverted face trials including the congruent and incongruent trials. If the performances of the participants are above chance, it means the part-based processing is observed.

## Experiment 1

**FIGURE 7:**
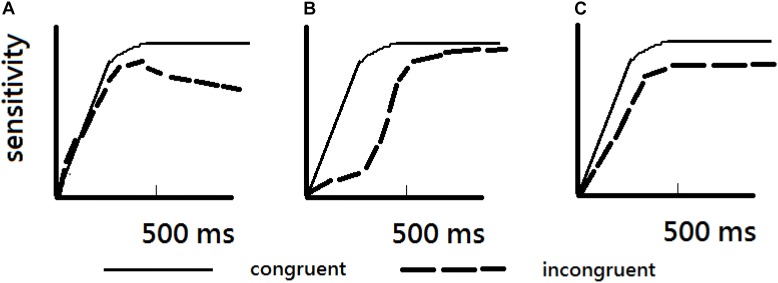
Richler et al. (2009) proposed three possibilities and their findings supported predictions depicted in **(C)**. Holistic processing, measured in terms of difference in performance between congruent and incongruent conditions, occurs at all exposure time when performances are above-chance performance. The figure was redrawn from Richler et al. (2009). **(A)** Illustrates that holistic processing occurs after a minimum of exposure time and increases as exposure duration was increased. **(B)** Illustrates that holistic processing is larger when exposure duration is limited. **(C)** Illustrates that holistic processing occurs without interference of exposure duration.

The issue of time course also concerns whether holistic processing may exist at a glance, and when it would disappear after a longer delay. Richler et al. (2009) proposed that holistic processing emerged very rapidly because they found that participants’ performance was above chance and did not depend on exposure time (see Figure 7). Note, however, their prediction was inconsistent with Hole’s (1994) findings, where he failed to find evidence of holistic processing at the duration of 2,000 ms; however, Richler et al. (2009) did not examine whether holistic processing occurs and still exists at the duration beyond 800 ms.

It should be noted, however, that there were inconsistent results regarding whether holistic processing was obtained with a relatively short term of duration. Therefore, in Experiment 1, a range of relatively brief exposure duration, namely 100, 200, and 500 ms, was manipulated to see whether or not can replicate these results for separation between holistic processing and part-based processing with these brief durations. In contrast, in Experiment 2, it manipulated relatively long exposure duration to see whether holistic processing still can be obtained with 1000 and 2000 ms. There has been a controversial issue whether holistic processing was obtained at 2000 ms. On one hand, Hole (1994) was unable to observe holistic processing at 2000 ms, but Richler et al. (2011) predicted that holistic processing should be obtained at 2000 ms according to their hypothesis (see Figure 7). In order to recreate the experimental setup as closely to that used by Hole (1994) as possible, a pair of faces was presented diagonally in each trial to reduce the possibility that participants might adopt a feature-by-feature comparison strategy to cope with the task. In addition, the whole study practically adopted a between-participant design and separate the two ranges of exposure duration in Experiments 1 and 2 as a means to provide a more conservative test of time course for part-based and holistic processing.

### Methods

#### Participants

Eighteen participants (9 male) from National Chung Cheng University participated in Experiment 1, and none of them had been exposed to the face composite task in the past 3 months. All participants had normal or corrected to normal vision, and each received a monetary payment of NTD$120 for their participation. It took about 55 min for participants to complete the experiment. Data from one participant had to be excluded because his overall performance was below chance. Likewise, the data from another participant was excluded because the performance was worse than two standard deviations from the mean. Participants were recruited in accordance with approval of the Research Ethics Committee of National Chung Cheng University, Chia-Yi, Taiwan (No. CCUREC104082101). All participants had completed the informed consents before the experiment.

#### Stimuli

For face stimuli, the present study first created 32 different Asian face images with equal number of males and females using *FaceGen 3.1* (Singular Inversions, Canada). Half of them, 8 males and 8 females, were chosen as the *relevant* set, and the remaining half, also 8 males and 8 females, was chosen as the *irrelevant* set. The top halves from the relevant set were randomly paired with the bottom halves from the irrelevant set for constructing face composites, in accordance with the requirement of complete design. Specifically, there were 16 faces for each of the four face composites designated as “A (top)/B (bottom),” “A (top)/C (bottom),” “D (top)/C (bottom),” and “D (top)/B (bottom)” (see Figure 6).

Each face image could be encircled by an (invisible) square with 75 pixels on each side. When presented on the display screen, each face was about 3.38 cm in width and 4.58 cm in height, extending a visual angle of about 3.01° × 3.6° at a viewing distance of approximately 45 cm. An overextended white line was overlaid horizontally in the mid-section of each face to clearly demark the top and bottom half of the face. The line was of 7.79 cm in length and 0.14 cm in height, extending a visual angle of 6.14° × 0.11°.

#### Design and Procedure

As mentioned above, in Experiment 1, the design manipulated a relatively brief range of exposure time of 100, 200, and 500 ms, as a within-participant variable. As in Experiment 1, it adopted the complete design (Figure 6) and computed differences in congruency effect between upright and inverted faces for each participant as the dependent variable (see section “A Note on Conceptual Clarification and the General Method”).

Each composite face was presented either upright or inverted for a total of 384 trials, comprising equal number of congruent and incongruent trials in accordance with the design used in Experiment 1. The upright face and inverted face conditions were separated into different blocks, the order of administration was counterbalanced across participants. In each trial, a “+” for fixation was first shown at the center of the display for 500 ms, followed by the presentation of a pair of composite faces, diagonally presented for one of the three durations, 100, 200, or 500 ms, where exposure time was manipulated as a within-participant variable. When the face stimuli was shown for the pre-designated exposure duration, a mask was presented for 200 ms. The manipulation of face orientation also was done as a within-participants variable by including two blocks of trials, one block with upright faces and the other with inverted faces. The order of the two blocks was counterbalanced across participants.

### Results

Mean percentage of correct identification, expressed in terms of hit rate (HR) and correction rejection (CR), of the top halves of upright and inverted conditions are shown in Table 1. The results show the same set of analyses as done in Experiment 1 as follows:

**Table 1:** Descriptive statistics for each condition in Experiment 1.

Orientation	Congruent	Response	Exposure duration		
			
			100 ms	200 ms	500 ms
Upright	Congruent	HR	0.50 (0.21)	0.55 (0.21)	0.86 (0.13)
		CR	0.59 (0.21)	0.66 (0.25)	0.86 (0.11)
		*d*′	0.24 (0.58)	0.78 (1.04)	2.42 (0.69)
	Incongruent	HR	0.40 (0.21)	0.46 (0.22)	0.72 (0.16)
		CR	0.65 (0.20)	0.67 (0.21)	0.74 (0.19)
		*d*′	0.18 (0.64)	0.44 (0.53)	1.57 (0.63)
Inverted	Congruent	HR	0.50 (0.21)	0.66 (0.17)	0.86 (0.14)
		CR	0.58 (0.25)	0.63 (0.17)	0.67 (0.23)
		*d*′	0.38 (0.73)	0.83 (0.52)	2.00 (0.91)
	Incongruent	HR	0.53 (0.22	0.62 (0.17)	0.84 (0.15)
		CR	0.54 (0.22)	0.60 (0.18)	0.66 (0.20)
		*d*′	0.18 (0.39)	0.57 (0.45)	1.71 (0.76)


*Means of hit rate (HR) and correct rejection (CR) for upright and inverted face condition at each level of exposure time. Standard deviations are shown in parentheses.*

#### Holistic Processing

**FIGURE 8:**
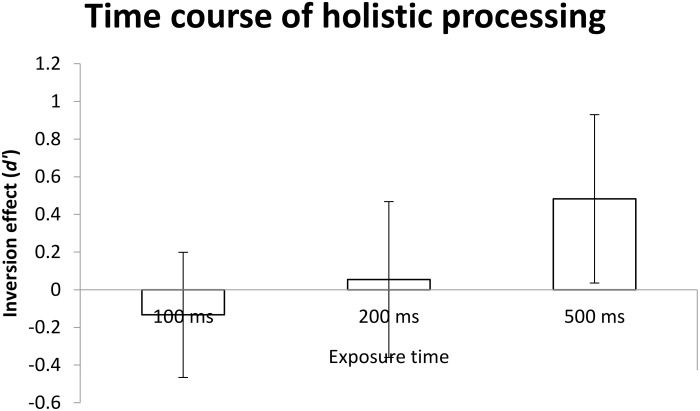
The mean *d*′ of inversion effect (i.e., the difference in *d*′ between upright and inverted conditions) as a function of exposure time. Error bars indicate 95% confidence interval, calculated within participants.

One-way measure ANOVA was conducted with exposure time (100, 200, and 500 ms) as the within-participant factor. As shown in Figure 8, the main effect of exposure time was not significant, *F*(2,32) = 3.08, *MSE* = 1.70, *p* > 0.05, $\eta_{p}^{2}$ = 0.161, indicating no difference in the magnitude of holistic processing as a function of exposure duration.

In terms of planned comparisons, one-sample *t*-tests and used Holm-Bonferroni method to control the family wise error rate (α_B_ = 0.017, α_B_ = 0.025, α_B_ = 0.05). were performed to test whether holistic processing would occur at each level of exposure duration. The results reveals that holistic processing was not observed at the level of two shorter exposure durations of 100 ms and 200 ms, *t*s < 1. However, holistic processing was obtained at exposure duration of 500 ms marginal significantly (*M* = 0.48), *t*(16) = 2.29, *p* = 0.036 > α_B_ = 0.017, 95% CI = [0.04, 0.93].

#### Part-Based Processing

The same one-way ANOVA was conducted with exposure time as the within-subjects factor, and *d*′ was the dependent measure for part-based processing. The main effect of exposure time was significant, *F*(2,32) = 103.63, *MSE* = 10.9, *p* < 0.001, $\eta_{p}^{2}$ = 0.87. *Post hoc* comparisons with Bonferroni correction (α_B_ = 0.017) revealed that performance at 500 ms was better than those at 100 and 200 ms, *ps* < 0.01 < α_B_s = 0.017), and performance at 200 ms in turn was better than that at 100 ms (*p* < 0.01 < α_B_s = 0.017). The results suggested that evidence of part-based processing emerged at about after 100 ms after stimulus onset and increased as more exposure time was available for further processing.

**FIGURE 9:**
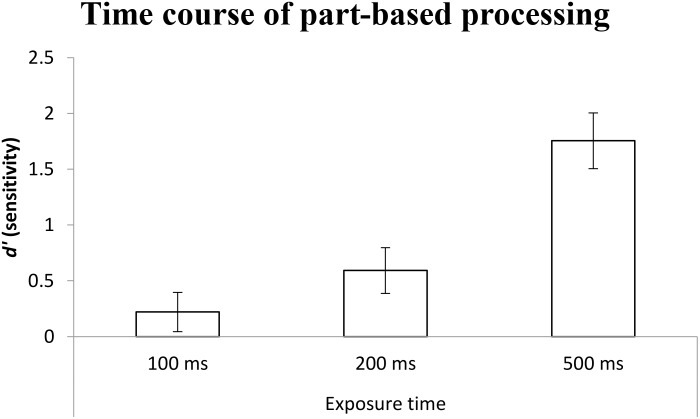
The mean *d*′ (sensitivity) for indexing part-based processing in each condition as a function of exposure time. Error bars indicate 95% confidence interval, calculated within participants.

In terms of planned comparisons, as shown in Figure 9, the results were again used one sample *t*-test and used Holm–Bonferroni method to control the family wise error rate (α_B_ = 0.017, α_B_ = 0.025, α_B_ = 0.05). The performances were above chance at all three levels of exposure time {100 ms: *t*(16) = 2.65, *p* = 0.017 < α_B_ = 0.05, *M* = 0.22, 95% CI = [0.04, 0.40]; 200 ms: *t*(16) = 6.13, *p* < 0.001 < α_B_ = 0.025, *M* = 0.59, 95% CI = [0.39, 0.80]; 500 ms: *t*(16) = 14.87, *p* < 0.001 < α_B_ = 0.017, *M* = 1.76, 95% CI = [1.51, 2.01]}. These results again indicate that part-based processing was in place very early on in processing faces after their presentation.

### Discussion

The results of Experiment 1, which offered strong support to the conjecture of separation between holistic processing and part-based processing, where the results also suggested that holistic processing occurred after part-based processing occurred. That is, the *d*′ results for each condition showed clear evidence for an early onset of part-based processing, whereas the inversion effect as evidence for holistic processing did not emerge until a longer exposure duration of 500 ms. These findings support to the time course of face processing depicted in Figure 7A. Alternatively, there is another possibility to explain the results where holistic processing and part-based processing occur simultaneously, but holistic processing may be prone to the disruption due to low stimulus discriminability as may well be the case in the face stimuli used in Experiment 1 because the means of the performances in the experiment were in general lower than the means of the studies from Richler et al. (2009, 2011). Even though the congruency effect between congruent and incongruent trials as Richler et al. (2009) did, the results still could not find the significant congruency effect at 100 and 200 ms (*t*s < 1.43 or *p*s > 0.1), but did find a significant congruency effect at 500 ms, *t*(16) = 5.17, *p* < 0.001.

## Experiment 2

As noted earlier, there has been an important and yet controversial issue regarding the time course of face processing. In order to examine whether the holistic processing of faces would sustain beyond the initial moment of detecting their presence, the present experiment used a simultaneous composite face task, where a pair of face stimuli were exposed for relatively lengthy duration of 500, 1000, or 2000 ms, respectively. With these durations, Hole (1994) had indicated that there was no difference between the upright and inverted faces at the long duration of 2000 ms. His result was inconsistent with the predictions made by Richler et al. (2009) (see Figure 7). However, Richler and her colleagues had not endeavored to demonstrate that holistic processing occurred after or beyond 800 ms. Therefore, Experiment 2 was designed to examine this controversy (Richler et al., 2009).

In order to have maximum opportunity to replicate Hole’s (1994) original findings, the present experiment adopted to display two face images in each trial diagonally to circumvent participants’ use of a feature-by-feature comparison strategy. In a pilot test, it was very difficult for participants to view two face images simultaneously when they were presented briefly for 500 ms, therefore adjusted stimulus size to make the face images easier to view and removed the mask that comes after showing two composite faces in Experiment 1, as Hole (1994) did. In addition, Experiment 2 adopted between-participant design regarding the manipulation of exposure duration to provide a more conservative test of the issue at hand.

### Methods

#### Participants

Fifty-eight college students from the National Chung Cheng University in Chiayi County, Taiwan, participated in the experiment. Of them, 21 (6 male, 15 female) were assigned to the exposure condition of 500 ms, 17 (6 male, 11 female) to 1000 ms, and 20 (7 male, 13 female) to 2000 ms, respectively. All participants had normal or corrected to normal vision, and each received a monetary payment of NTD$100 for their participation. It took about 30 min for participants to complete the experiment. As experiment 1, participants were recruited in accordance with approval of the Research Ethics Committee of National Chung Cheng University, Chia-Yi, Taiwan (No. CCUREC104082101). Each participant had completed the informed consents when began the experiment.

#### Stimuli and Apparatus

The stimuli were the same as Experiment 1, except that each face image was made slightly bigger, which could be encircled by an (invisible) square with 100 pixels on each side. When presented on the display screen, each face had a width of about 5.1 cm and a height of about 6.1 cm, extending a visual angle of about 4.01° × 4.8° at a viewing distance of approximately 45 cm.

A pair of faces were simultaneously presented on the display for a pre-determined duration of 500, 1,000, or 2,000 ms. Unlike Experiment 1 where face stimuli were presented at the same horizontal height, one face was located in the upper left quadrant and the other face was located in the lower right quadrant to discourage feature-by-feature comparisons. The center of the face located in left quadrant was about 4.69 cm below the top edge of the monitor, and about 13.31 cm to the right of left edge of the monitor. The center of the face located in the lower-right quadrant roughly had the same distances from the bottom and right edge of the monitor. The two faces were separated by a center-to-center distance of about 14.12 cm.

#### Design and Procedure

The same design used in Experiment 1 was used in Experiment 2, except for the exact length of exposure duration and the nature of manipulation. Specifically, three levels of relatively long exposure duration of 500, 1000, and 2000 ms was varied as a between-participant factor. As in Experiment 1, the design of the present experiment is the complete design and computed differences in congruency effect between upright and inverted faces for each participant as the dependent measure for holistic processing.

#### Procedure

Each composite face was presented either upright or inverted for a total of 128 trials, with equal number of congruent and incongruent trials in accordance with the complete design. The upright face and inverted face conditions were separated into different blocks and the order of administration was counterbalanced across participants. In each trial, a “+” for fixation was first shown at the center of the display for 500 ms, followed by the presentation of a pair of composite faces for one of the three durations, 500, 1000, or 2000 ms, where exposure times was manipulated as a between-participants variable. Upon seeing the face pair, participants were asked to judge whether the top halves of the two faces were identical, while ignoring the bottom halves. The manipulation of face orientation was done as a within-participants variable by including two blocks of trials, one block with upright faces and the other with inverted faces. The order of administering the two blocks was counterbalanced across participants.

### Results

The mean accuracy for correct judgment of the top halves of upright and inverted conditions, in terms of hit rate (HR) and correct rejection (CR) and *d*′ derived from them, are shown in Table 2.

**Table 2:** Descriptive statistics for each condition in Experiment 2.

Orientation	Congruent	Response	Exposure duration		
			
			100 ms	200 ms	500 ms
Upright	Congruent	HR	0.68 (0.14)	0.86 (0.12)	0.89 (0.13)
		CR	0.80 (0.11)	0.86 (0.12)	0.90 (0.16)
		*d*′	1.51 (0.75)	2.59 (0.89)	3.03 (1.14)
	Incongruent trials	HR	0.61 (0.19)	0.7 (0.12)	0.71 (0.18)
		CR	0.67 (0.17)	0.77 (0.14)	0.82 (0.11)
		*d*′	0.82 (0.67)	1.36 (0.54)	1.60 (0.96)
Inverted	Congruent trials	HR	0.62 (0.16)	0.76 (0.17)	0.83 (0.14)
		CR	0.76 (0.17)	0.81 (0.11)	0.81 (0.13)
		*d*′	1.63 (1.32)	1.84 (0.53)	2.17 (0.77)
	Incongruent trials	HR	0.57 (0.14)	0.71 (0.15)	0.77 (0.15)
		CR	0.74 (0.14)	0.76 (0.13)	0.83 (0.09)
		*d*′	0.87 (0.46)	1.36 (0.54)	1.85 (0.65)


*Means of hit rate (HR) and correct rejection (CR) for upright and inverted face condition in 500, 1000, and 2000 ms, respectively. Standard deviations are shown in parentheses*.

#### Holistic Processing

**FIGURE 10:**
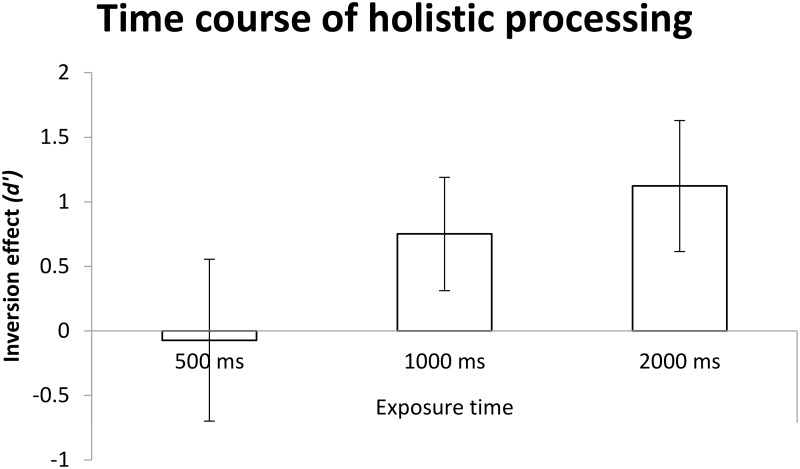
The mean *d*′ for dependent variable as a function of exposure time. Error bars indicate 95% confidence interval, calculated within participants.

A one-way ANOVA was conducted with exposure duration as the sole between-participants factor and its main effect of exposure time was significant, *F*(2,55) = 5.87, *MSE* = 7.67, *p* < 0.01, $\eta_{p}^{2}$ = 0.176. *Post hoc* comparisons with Bonferroni correction for multiple comparison (α_B_ = 0.017) revealed that, as shown in Figure 10, the magnitude of holistic processing at 2000 ms was greater than that at 500 ms, *p* = 0.001 < α_B_ = 0.017; no other comparisons were found significant *p*s > α_B_ = 0.017, however.

In terms of planned comparisons, one-sample *t*-tests were performed, and used Holm–Bonferroni method to control the family wise error rate (α_B_ = 0.017, α_B_ = 0.025, α_B_ = 0.05). The results showed holistic processing was not observed at the exposure duration of 500 ms, *t* < 1, but was found at both 1000 and 2000 ms {1000 ms: *t*(16) = 3.63, *p* < 0.01 < α_B_ = 0.025, *M* = 0.75, 95% CI = [0.31, 1.19]; 2000 ms: *t*(19) = 4.63, *p* < 0.001 < α_B_ = 0.017, *M* = 1.12, 95% CI = [0.61, 1.63]}.

#### Part-Based Processing

**FIGURE 11:**
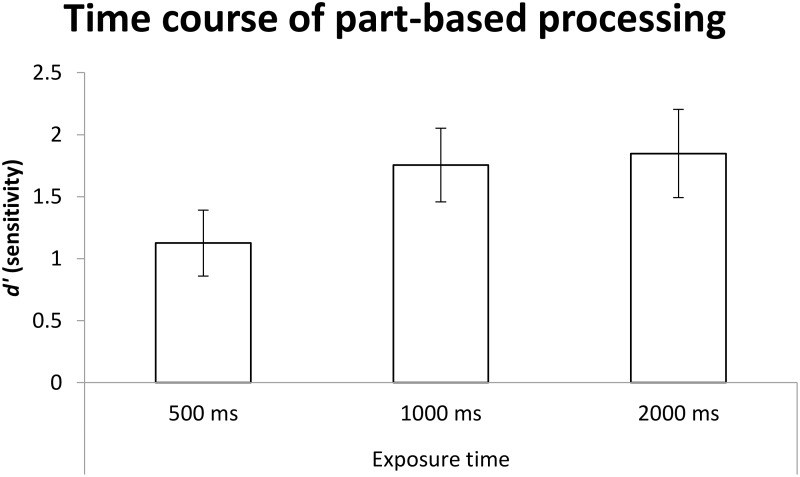
The mean *d*′ for sensitivity as a function of exposure time. Error bars indicate 95% confidence interval, calculated within participants.

The same one-way ANOVA was conducted, with exposure duration as the between-participants factor, and as for holistic processing, its main effect was significant, *F*(2,55) = 7.42, *MSE* = 3.14, *p* < 0.01, $\eta_{p}^{2}$ = 0.213. *Post hoc* comparisons with Bonferroni correction for multiple comparison (α_B_ = 0.017), as shown in Figure 11, revealed that performance at 500 ms was worse than those at 1000 ms (*p* = 0.013 < α_B_ = 0.017) and 2000 ms (*p* < 0.01 < α_B_ = 0.017), and there was no significant difference between the latter two conditions (*p* > α_B_ = 0.017).

In terms of planned comparisons, performances were better than chance at all three levels of exposure duration {500 ms: *t*(20) = 8.81, *p* < 0.001 < α_B_ = 0.017, *M* = 1.13, 95% CI = [0.86, 1.39]; 1000 ms: *t*(16) = 12.51, *p* < 0.001 < α_B_ = 0.017, *M* = 1.75, 95% CI = [1.46, 2.05]; 2000 ms: *t*(19) = 10.85, *p* < 0.001 < α_B_ = 0.017, *M* = 1.84, 95% CI = [1.49, 2.20]}.

### Discussion

In Experiment 2, holistic processing clearly is observed at 1000 ms and lasted till 2000 ms and perhaps even beyond. While this finding was consistent with the prediction made by Richler et al. (2009), it remains to be explained why the early study by Hole (1994) failed to find the composite face effect when participants saw two composite faces simultaneously with 2000 ms of exposure duration. There are three possibilities for the discrepancy: First, there was a clear difference regarding the nature of task and its design, where the present study was based on the complete design, but Hole (1994) adopted the traditional partial design.

**FIGURE 12:**
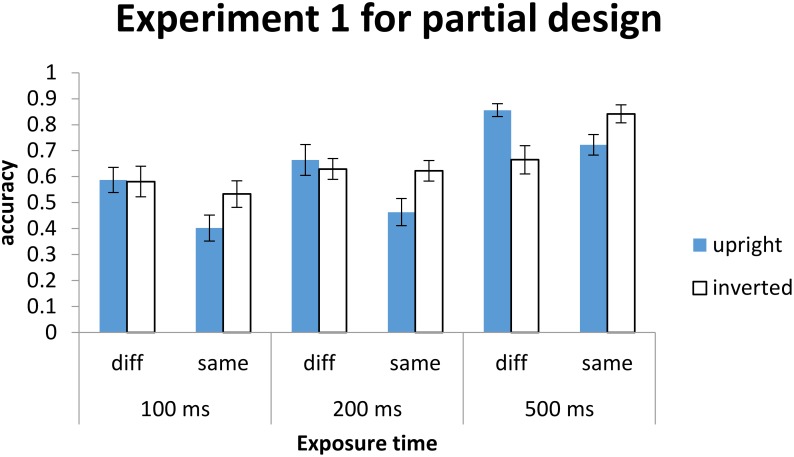
The meanaccuracy for response as a function of orientation for exposure time. Error bars indicate ±1 SE, calculated within participants.

**FIGURE 13:**
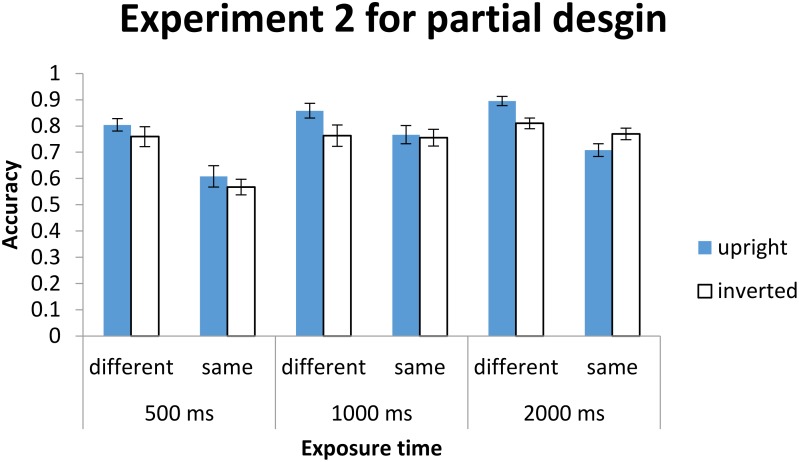
The mean accuracy for response as a function of orientation for exposure time. Error bars indicate ±1 SE, calculated within participants.

Second, another possibility is the indices Hole chose were different from those chosen in the present study. Specifically, as measuring differences in congruency effect between upright and inverted condition, as proposed by Gauthier and Bukach (2007); in contrast, Hole (1994) used inversion superiority effect. Although Hole calculated reaction time to measure face inversion superiority, the results did not show reaction time (RT) because was not very useful to analyze reaction time data when the mean accuracy in a number of conditions were below 70% (Tables 1, 2), which may well lead to biased estimate of RT performances. However, the results did find inversion superiority effect based on accuracy for exposure duration in100 ms (see Figure 12 and Appendix 1), but failed to do so for exposure duration from 200 to 500 ms in Experiment 1 and 500 to 2000 ms in Experiment 2 (see Figure 13 and Appendix 2). In other words, it is relatively easy to detect inversion superiority effect with short exposure durations than with longer exposure durations.

Finally, according to Flin (1985) which suggested that stimuli discrimination may interfere with inversion effect. The mean accuracy for participants’ performances was higher than 99% in the early study by Young et al. (1987), and it was similarly high in Hole’s (1994) study (i.e., 88%). These results suggest that the faces (and their composites) used in the earlier study were relatively easy for participants to judge, and if the composite faces were too hard to judge, inversion effect might not be obtained at all. As Flin had remarked, inversion effect cannot be detected if there were floor (or ceiling) effect, which points out the possibility that perceptual discriminability of face stimuli may modulate the presence as well as strength of holistic processing. However, there has been very little research to investigate systematically how perceptual discriminability may affect holistic processing.

## General Discussion

Taken together, the results from Experiments 1 and 2, revealed the separation between holistic processing and part-based processing, and as such gave credence to the dual-route model on face recognition envisioned by McKone (2010).

It is also interesting to note that at 500 ms of exposure duration, holistic processing was found in Experiment 1, but not in Experiment 2. There are some possibilities, for example, one possible explanation for the discrepancy is that holistic processing could be under the influence of spatial distance between a pair of stimulus faces. Another explanation is the design and the mask to cause the differences. In the future, it needs to be further manipulated for this issue.

In summary, the results of Experiments 1 and 2 indicated that holistic processing was maintained until 2000 ms, supporting the prediction by Richler et al. (2009). However, it is not supported that holistic processing would automatically occurred at part-base performance that was above chance. According to the third hypothesis (Figure 7) from Richler et al. (2009), they predicted that congruency effects are obtained as above-chance performance (of part-based processing) is observed and their results reveals that congruency effects were obtained in 50 ms to 800 ms when the performances were better than chance. In contrast, the results suggested that even when part-based performance was better than chance, holistic processing did not occur automatically. For example, with exposure durations of 1000 and 2000 ms, holistic processing did occur. Rather, it seems that sufficient exposure duration is a key factor for producing evidence for holistic processing. In addition to exposure duration, if the spatial distance between two face stimuli was too great to see the pair of faces simultaneously, holistic processing may also be compromised. In the future, it is necessary to investigate systematically how perceptual discriminability and spatial factor may affect holistic processing.

## Author Contributions

C-CW contributed to the rationale of the whole experiments, analyzed the data, and drafted the manuscript.

## Conflict of Interest Statement

The author declares that the research was conducted in the absence of any commercial or financial relationships that could be construed as a potential conflict of interest. The reviewer YU and handling Editor declared their shared affiliation.

## Appendix

### Appendix 1: Experiment 1

There was a 2 × 2 × 3 repeated-measured ANOVAs conducted with Orientation (upright/inverted), Exposure time (100, 200, and 500 ms) and Response (same/different) as within-participant variables. A significant main effect of exposure time was found, *F*(2,34) = 92.2, *MSE* = 1.15, *p* < 0.001, $\eta_{p}^{2}$ = 0.844, which was modulated by two-way interaction with orientation, *F*(2,34) = 5.14, *MSE* = 0.058, *p* < 0.05, $\eta_{p}^{2}$ = 0.232. A simple main effect revealed that the performance of inverted faces (*M* = 0.56) was better than that of upright faces (*M* = 0.49) in 100 ms (*p* < 0.01), but there were no significant differences in 200 and 500 ms (*p*s > 0.05). As a result, inversion superiority effect was found in 100 ms. There was a significant two-way interaction between orientation and response, *F*(1,17) = 23.23, *MSE* = 0.61, *p* < 0.001, $\eta_{p}^{2}$ = 0.577. The difference between accuracy of different and same trials in the upright faces (*M* = 0.17) was greater than that in the inverted faces (*M* = -0.04). No other main effects and interactions were found.

### Appendix 2: Experiment 2

The mean accuracy in each condition was first submitted to a three-way mixed-measured analysis of variance (ANOVA) with face orientation (upright vs. inverted) and response (same vs. different) as within-participant variables and exposure duration (500, 1000, and 2000 ms) as between-participants variable. The results showed that performance on upright trials (*M* = 0.77) was better than that on inverted trials (*M* = 0.74), as reflected in a significant main effect of orientation, *F*(1,55) = 9.19, *MSE* = 0.07, *p* < 0.001, $\eta_{p}^{2}$ = 0.143. However, inversion superiority effect was not found. There was also a main effect of response, *F*(1,55) = 26.07, *MSE* = 0.82, *p* < 0.001, $\eta_{p}^{2}$ = 0.322. A main effect of exposure time was found [*F*(1,55) = 9.92, *MSE* = 0.31, *p* < 0.001, $\eta_{p}^{2}$ = 0.265]. Although two-way interaction between orientation and response was found, *F*(1,55) = 4.15, *MSE* = 0.89, *p* < 0.05, $\eta_{p}^{2}$ = 0.07, the difference between accuracy of different trials and same trials in the upright faces (*M* = 0.16) was greater than that of the inverted face (*M* = 09). The present study also reveals the two-way interaction between exposure time and response [*F*(2,55) = 3.19, *MSE* = 0.10, *p* < 0.05, $\eta_{p}^{2}$ = 0.104], but two-way interaction between orientation and exposure time was not found. No three-way interaction was found [*F*(1,55) = 1.22, *MSE* = 0.026, *p* > 0.05]. According to these results, inversion superiority effect cannot be obtained.
